# Environmental Free-Living Amoebae Can Predate on Diverse Antibiotic-Resistant Human Pathogens

**DOI:** 10.1128/AEM.00747-21

**Published:** 2021-08-26

**Authors:** Félix Bornier, Eline Zas, Damien Potheret, Maria-Halima Laaberki, Bénédicte Coupat-Goutaland, Xavier Charpentier

**Affiliations:** a Centre International de Recherche en Infectiologie, Team Horizontal Gene Transfer in Bacterial Pathogens, INSERM U1111, Université Claude Bernard Lyon 1, CNRS UMR 5308, École Normale Supérieure de Lyon, Université de Lyon, Villeurbanne, France; b Université de Lyon, VetAgro Sup, Marcy l'Etoile, France; Centers for Disease Control and Prevention

**Keywords:** amoebae, bactericidal activity, Gram-negative bacteria, host-pathogen interactions

## Abstract

Here, we sought to test the resistance of human pathogens to unaltered environmental free-living amoebae. Amoebae are ubiquitous eukaryotic microorganisms and important predators of bacteria. Environmental amoebae have also been proposed to serve as both potential reservoirs and training grounds for human pathogens. However, studies addressing their relationships with human pathogens often rely on a few domesticated amoebae that have been selected to feed on rich medium, thereby possibly overestimating the resistance of pathogens to these predatory phagocytes. From an open-air composting site, we recovered over 100 diverse amoebae that were able to feed on Acinetobacter baumannii and Klebsiella pneumoniae. In a standardized and quantitative assay for predation, the isolated amoebae showed a broad predation spectrum, killing clinical isolates of A. baumannii, K. pneumoniae, Pseudomonas aeruginosa, and Staphylococcus aureus. Interestingly, A. baumannii, which was previously reported to resist predation by laboratory strains of *Acanthamoeba*, was efficiently consumed by closely related environmental amoebae. The isolated amoebae were capable of feeding on highly virulent carbapenem-resistant or methicillin-resistant clinical isolates. In conclusion, the natural environment is a rich source of amoebae with broad-spectrum bactericidal activities, including against antibiotic-resistant isolates.

**IMPORTANCE** Free-living amoebae have been proposed to play an important role in hosting and disseminating various human pathogens. The resistance of human pathogens to predation by amoebae is often derived from *in vitro* experiments using model amoebae. Here, we sought to isolate environmental amoebae and to test their predation on diverse human pathogens, with results that challenge conclusions based on model amoebae. We found that the natural environment is a rich source of diverse amoebae with broad-spectrum predatory activities against human pathogens, including highly virulent and antibiotic-resistant clinical isolates.

## INTRODUCTION

Amoebae are unicellular eukaryotic microorganisms that are found in natural aquatic and terrestrial environments in temperate climates but also in more extreme environments such as polar melt water, arid land, and tropical forests (1, 2). These protists move and feed by emitting cytoplasmic extensions called pseudopods. While some, such as Entamoeba histolytica, are parasitic (3), others that do not depend on a host are classified as free-living (1). Amoebae have at least a two-step life cycle, i.e., the trophozoite form and the cyst form (4). The trophozoite form is a vegetative state during which the cells are metabolically active, move, feed, and reproduce (4). Under adverse environmental conditions (such as osmotic stress, temperature, pH, predators, or antagonistic compounds), the amoebae can adopt a quiescent form, the cyst, in order to persist in their environment. Once favorable conditions return, they can excyst back to the trophozoite form. In the environment, amoebae graze naturally on bacteria, fungi, or other protists, which they engulf by phagocytosis into digestive vacuoles (1, 5).

Thus, amoebae are predators that naturally regulate populations of multiple microorganisms in the environment and play an important ecological role (5, 6). However, the relationships of amoebae and bacteria are complex and can extend to mutualistic and parasitic interactions (2, 7). While predatory interactions can have ecological implications (8), parasitic interactions have attracted considerable interest in biomedical research. Indeed, some parasitic amoeba-resistant bacteria are also human pathogens (9), highlighting a role of amoebae as reservoirs and/or means of dissemination (10) but also as a training ground for pathogens (11). The latter hypothesis primarily resulted from the observation that amoebae could support the growth of the human pathogen Legionella pneumophila (12) and that this pathogen uses the same mechanisms to survive and to replicate in human macrophages (13). It is now well established that amoebae are true natural hosts and reservoirs of *Legionella* and that grazing by these environmental predators has selected for traits that are also required to infect and to kill human phagocytes (14). Beyond L. pneumophila, it has been proposed that adaptation and resistance to trophic interactions can select for traits that can contribute to pathogenesis in mammalian hosts (15). Accordingly, well-characterized and easy-to-grow amoebae, such as Acanthamoeba castellanii and the social amoeba Dictyostelium discoideum, have emerged as models to identify factors contributing to virulence in mammalian hosts (16). For instance, they allowed the identification of the now highly studied type VI secretion system (17) and the contribution of the capsule of Klebsiella pneumoniae to resistance to phagocytosis by D. discoideum, which also plays a role in resistance to human neutrophils (18). A number of studies used the same domesticated amoebae to test the hypothesis that human pathogens, such as Helicobacter pylori, Vibrio cholerae, pathogenic Escherichia coli, Mycobacterium avium, Listeria monocytogenes, Staphylococcus aureus, and Coxiella burnetii, could resist predation by related environmental amoebae (19–32). While H. pylori could indeed be recovered from natural isolates of amoebae (33), associations of most other pathogens with amoebae in natural environments are often not available (2). Studies aimed at finding associations of amoebae and bacteria in natural settings have reported that *Legionella*, Mycobacterium, Chlamydia, and Pseudomonas species are the most common bacteria found coexisting with amoebae (2, 34). It may be that the use of domesticated amoebae, which have been selected to feed on liquid medium rather than on bacteria, tends to overestimate the resistance of other pathogens to amoebae in the environment. Owing to the difficulties of identifying negative associations in natural settings, the ability of amoebae to predate on human pathogens is possibly underestimated.

In this study, we aimed to test the hypothesis that environmental amoebae can predate on a wide range of human pathogens, including multidrug-resistant strains. We used a culture-based approach to obtain undomesticated amoebae based on their capacity to feed on specific species, and we characterized their predatory activity against other bacterial species and diverse clinical isolates harboring distinct virulence traits and often resistance to antibiotics. Amoebae predating on human pathogens were found in five genera, *Tetramitus*, *Acanthamoeba*, *Vermamoeba*, *Vahlkampfia*, and *Stemonitis*. Interestingly, pathogens previously reported as resisting predation by domesticated amoebae were found to be consumed by natural isolates of the same amoebal genera/species. Most amoebae could predate on multiple pathogens (Acinetobacter baumannii, Klebsiella pneumoniae, Pseudomonas aeruginosa, and Staphylococcus aureus), regardless of presumed virulence traits or antibiotic resistance. Specifically, one isolate presented high levels of predatory activity against all tested pathogens, decimating outnumbering antibiotic-resistant bacterial populations by up to 6 orders of magnitude in 24 h. This study documents the characteristics of trophic interactions between diverse undomesticated amoebae and bacteria. Voracious amoebae with high levels of predatory activity may represent an untapped resource to control and fight populations of antibiotic-resistant pathogens.

## RESULTS

### Isolation and identification of amoebae feeding on multidrug-resistant bacteria.

**FIG 1:**
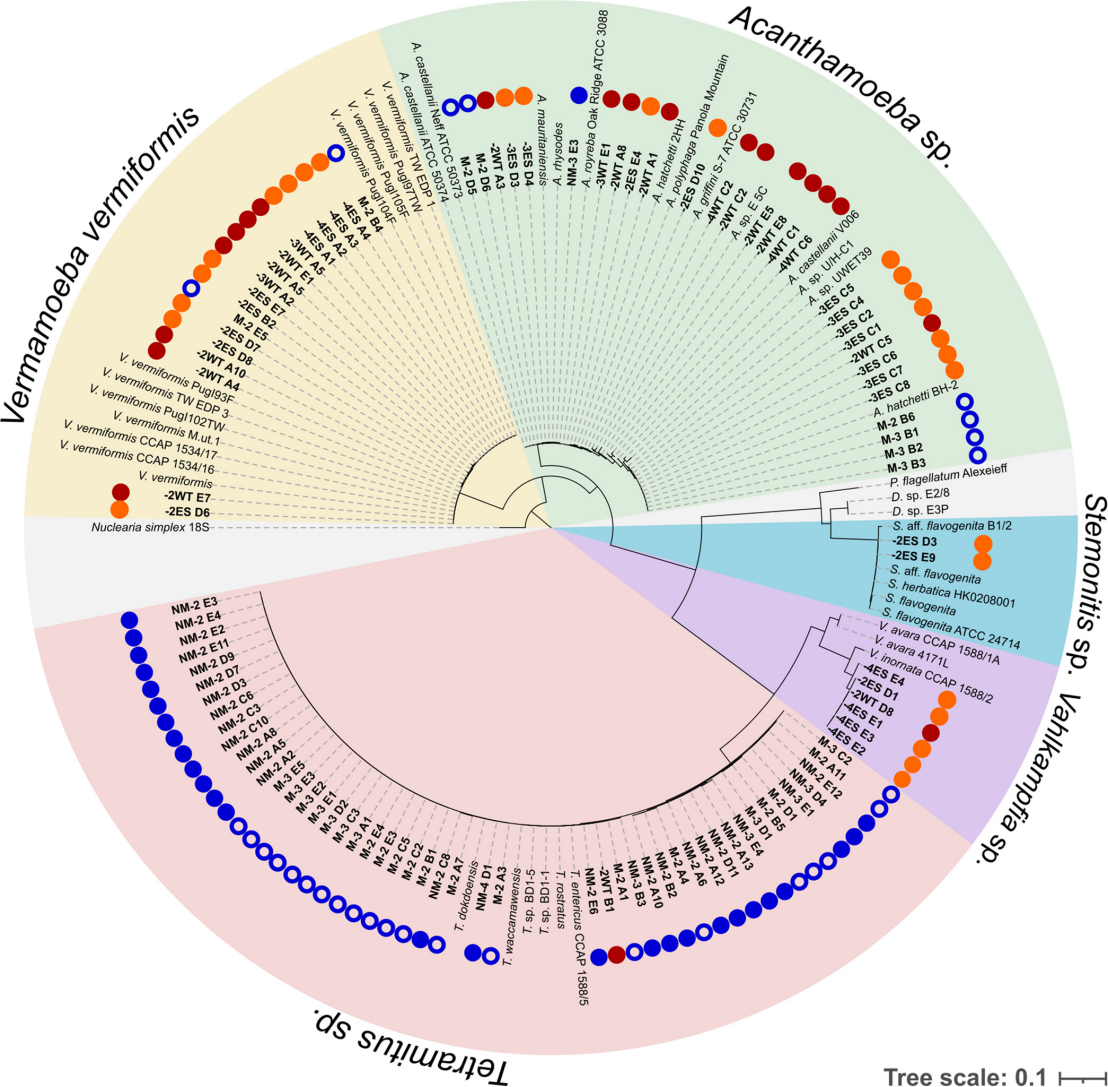
Phylogeny of 104 amoebae isolated from a composting site using A. baumannii strains (blue circles) or K. pneumoniae strains (red and orange circles) as a food source. The partial SSU rDNA tree was inferred with the maximum likelihood approach (437 comparison sites) for amoebae isolated from environmental samples. Isolated amoebae are indicated in bold. Amoebae isolated on A. baumannii AB5075F (solid blue circles) and the constitutively capsulated mutant AB5075F-M (open blue circles) are labeled NM and M, respectively. Amoebae isolated on K. pneumoniae zt246 and the ESBL-producing K. pneumoniae 26425 are labeled WT and ES, respectively. Reference sequences of different amoebal genera are written in black. The accession numbers of reference sequences and the sequences of the isolates of this study are available in Tables S1 and S2, respectively, in the supplemental material. *Nuclearia simplex* (Opisthokonta) was used as the outgroup. The scale bar shows the fraction of substitutions per site.

We set out to isolate amoebae based on their ability to feed on two multidrug-resistant bacterial human pathogens, A. baumannii and K. pneumoniae. Two K. pneumoniae clinical isolates were selected, strain zt246 and 26425, with the latter producing an extended-spectrum β-lactamase (ESBL). A. baumannii strain AB5075 is a clinical isolate that is resistant to carbapenems. In order to facilitate microscopic observation, we genetically modified it to produce the superfolder green fluorescent protein (GFP); here, it is referred to as AB5075F. In order to test a possible role of capsule production in resistance to amoebal predation (18), we obtained a strain with a spontaneous mutation in the *wzc* gene, here designated AB5075F-M, which forms highly mucoid colonies and constitutively expresses a thick capsule (see Fig. S1 in the supplemental material). The two K. pneumoniae isolates and the two A. baumannii strains were then used as food source to isolate amoebae from a compost sample. In a first isolation campaign, suspensions of the diluted compost samples were deposited on nonnutritive agar (NNA) plates coated with either the parental or mucoid strains. This resulted in the isolation of 57 amoebae with the ability to grow using A. baumannii as their sole nutrient source (Fig. 1, open and solid blue circles). Of these, 28 were isolated in the presence of the A. baumannii AB5075F strain (Fig. 1, solid blue circles), while the remaining 29 were obtained with the constitutively capsulated mutant AB5075F-M (Fig. 1, open blue circles). Following the same procedure, a second isolation campaign using another sample from the same composting site led to the isolation of 47 amoebae capable of growing using K. pneumoniae as the only nutrient source. Of these, 20 were isolated in the presence of the K. pneumoniae strain zt246 (Fig. 1, red circles), while the other 27 were isolated with the ESBL-producing K. pneumoniae strain 26425 (Fig. 1, orange circles). All 104 amoebal isolates were purified, and phylogeny and taxonomic attribution was performed using the sequence of the 18S small subunit (SSU) rRNA region (Fig. 1).

In all, the amoebae belonged to five genera, *Tetramitus*, *Acanthamoeba*, Vermamoeba vermiformis, *Vahlkampfia*, and *Stemonitis* (Fig. 1). Amoebae identified as *Tetramitus* sp. were distributed around Tetramitus entericus, Tetramitus rostratus, Tetramitus dokdoensis, and Tetramitus waccamawensis (formerly Learamoeba waccamawensis) (35) and may belong to different and possibly new species. Amoebae belonging to the *Tetramitus* genus have been isolated from aquatic or soil environmental samples but remain poorly studied (36–38). Because it is one of the most frequently isolated amoebal genera in the environment, we were not surprised to isolate a large number *Acanthamoeba* isolates (39). Isolates clustered around different genotypes defined by the hypervariable regions found in the 18S SSU rRNA gene (40, 41) and appeared diverse, grouping with genotypes T2, T3, T4, and T11 (Fig. 1). Amoebae isolated and identified as Vermamoeba vermiformis appeared less diverse, and this genus includes only one species (42, 43). This species has been frequently isolated from aquatic environments and soil samples, as well as a compost facility (38, 42, 44). The amoebae identified as *Vahlkampfia* grouped closer to the species Vahlkampfia inornata than to Vahlkampfia avara. Like *Tetramitus*, this amoebal genus has been isolated from environmental samples but remains poorly described and studied (35, 45, 46). Two amoebae were identified as *Stemonitis* (formerly *Hyperamoeba*), amoebae that are close to slime molds and whose phylogeny has long been a source of debate (47, 48). Also, but not displayed in the tree of Fig. 1, we isolated one ciliate (*Telotrochidium* sp.) feeding on A. baumannii, two ciliates belonging to the *Kreyellidae* family and *Colpoda* genus, and one kinetoplastid microorganism (*Dimastigella* sp.) feeding on K. pneumoniae strains. *Tetramitus* is the amoebal genus that was most frequently isolated in the presence of the A. baumannii AB5075F strain (96%), followed by the *Acanthamoeba* genus (4%). The Vermamoeba vermiformis amoebal species was isolated only in the presence of the mucoid mutant AB5075F-M. *Tetramitus* and *Acanthamoeba* amoebae were isolated in similar proportions on the wild-type strain and the constitutively capsulated mutant of AB5075 (Fig. 1). *Acanthamoeba* is the amoebal genus that was most frequently isolated on both strains of K. pneumoniae, followed by V. vermiformis. While the *Tetramitus* genus was dominant in the isolates feeding on A. baumannii, only one amoeba belonging to this genus was isolated on K. pneumoniae. In contrast, the isolation campaign on K. pneumoniae uncovered genera not found on A. baumannii. Six amoebae from the *Vahlkampfia* genus were isolated on both K. pneumoniae strains, and two amoebae, identified as *Stemonitis* sp., were isolated on K. pneumoniae zt246.

In all, we isolated 104 amoebae belonging to five genera, three ciliates, and one kinetoplastid flagellate capable of feeding on either A. baumannii or K. pneumoniae. This indicates that diverse amoebae and protists can use these pathogens as food sources. Indeed, even if the isolations on A. baumannii and K. pneumoniae were conducted independently, many amoebae isolated on one pathogen were phylogenetically undistinguishable from amoebae isolated on the other pathogen (Fig. 1). This suggests that some of the isolated amoebae would be able to feed on both species. Also, amoebae isolated on the wild-type strain and the capsulated mutant were often found within the same clade, suggesting that the constitutive production of a thick capsule by the prey did not select specific amoebae.

### Kinetics of amoebal predation on A. baumannii are not altered by constitutive expression of a thick capsule.

**FIG 2:**
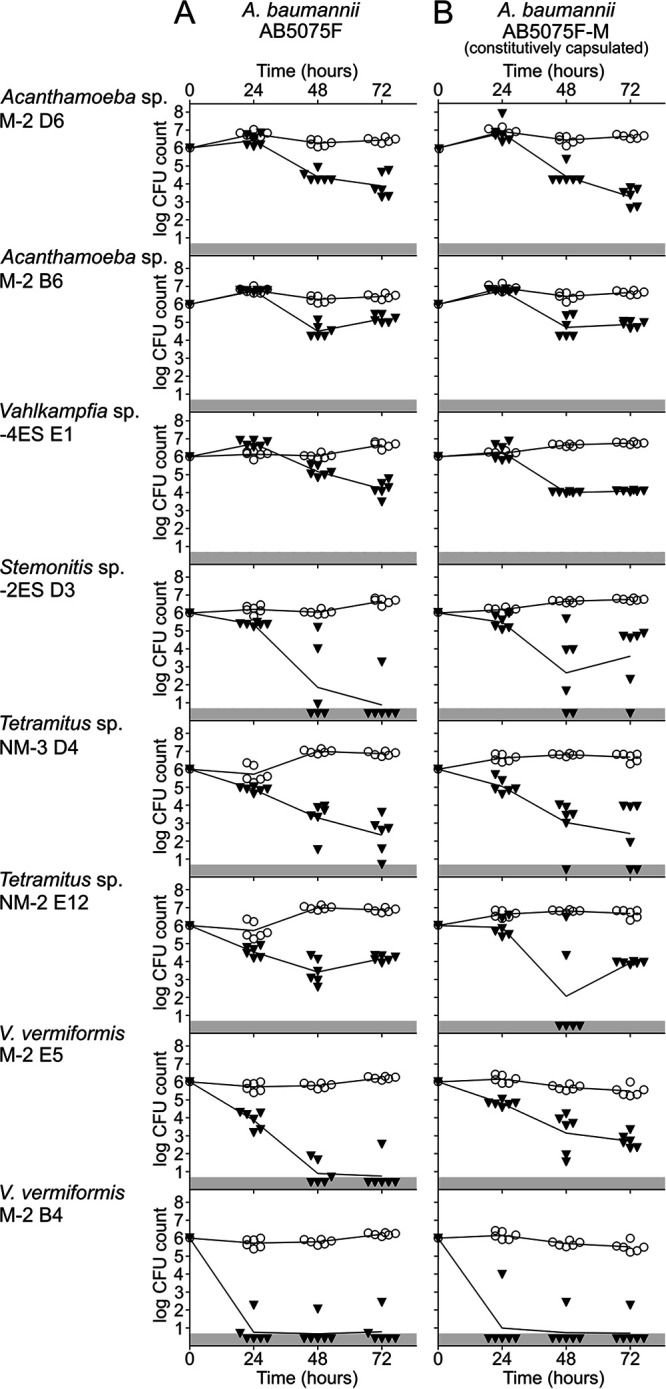
Predation activity of selected isolated amoebae. Amoebal isolates selected on A. baumannii AB5075F (NM), the capsulated AB5075F-M (M), or K. pneumoniae 26425 (ES) were tested for their ability to prey on A. baumannii AB5075F and the capsulated AB5075F-M. The size of the initial bacterial population (∼10^6^ bacteria) of AB5075F (A) or the capsulated AB5075F-M (B) was determined by CFU counting in the absence (open circles) or presence (black inverted triangles) of selected amoebae. CFU counts at time zero are based on enumeration of the bacteria deposited in the well. CFU counts at 24, 48, and 72 h correspond to the number of bacteria recovered from the wells. If no CFU were detected, then the sample was given the value of the detection limit (5 CFU) (gray area), corresponding to the number of CFU possibly present in the nonplated fraction of the collected sample.

We then sought to characterize the predation activity of eight arbitrarily selected amoebae within the five isolated genera, i.e., two amoebae from the *Acanthamoeba* and *Tetramitus* genera, two from the V. vermiformis species, and one from the *Stemonitis* and *Vahlkampfia* genera. In order to compare the activity of different amoebae and bacteria, we established a standardized protocol to monitor the effect of predation on populations of bacteria in excess, relative to the number of amoebae. Amoebae were cultivated on lawns of nonpathogenic E. coli K-12, harvested, and starved to induce their development into cysts. About 1 × 10^5^ cysts were then exposed to 1 × 10^6^ bacteria (here, the A. baumannii wild-type strain) on NNA. In the absence of amoebae, this medium allowed the bacterial population size to slightly increase and remain steady for 72 h (Fig. 2A, open circles). In contrast, in the presence of all eight tested amoebae, this initial AB5075F population was contained or even fell below the detection limit, with kinetics that differed between the tested amoebae (Fig. 2A, black inverted triangles). The two tested *Acanthamoeba* isolates and *Vahlkampfia* 4ES E1 could not alter the A. baumannii population during the first 24 h. However, they were able to consume it during the next 48 h, so that the A. baumannii population was about 10- to 100-fold lower than if it had not been exposed to amoebae. Two tested *Tetramitus* amoebae were effective more rapidly, being able to alter the population at 24 h and to steadily consume it, reducing the original population by 3 to 4 orders of magnitude. The *Stemonitis* 2ES D3 was also capable of containing the A. baumannii population at 24 h and then consumed it rapidly, resulting in a reduction of the population by over 4 orders of magnitude at 72 h. The two isolated V. vermiformis amoebae showed both stronger and faster bactericidal activity, with a moderate (M-2 E5) to strong (M-2 B4) reduction in the A. baumannii population at 24 h, leading to a dramatic reduction of the population that fell below the detection limit within 48 h. The same amoebae, which were isolated on K. pneumoniae, wild-type A. baumannii, or constitutively capsulated A. baumannii, were then tested against the constitutively capsulated AB5075F-M strain (Fig. 2B, black inverted triangles). V. vermiformis M-2 E5 seemed to reduce the bacterial population less efficiently but still managed to reduce it by over 2 orders of magnitude. For all other amoebae, the kinetics of bacterial population reductions were largely similar to those observed with the wild-type A. baumannii AB5075F (Fig. 2A, black inverted triangles). We conclude that, under the tested conditions, constitutive expression of a thick capsule offered little to no protection to predation by undomesticated amoebae. All tested amoebae proved effective at controlling and killing bacterial populations of A. baumannii but with large differences in the extent and kinetics of control; these may be due to distinct morphological properties, trophic activity, excystation rates, and/or production of bactericidal compounds.

### Bactericidal activity of amoebae results from trophic interaction.

**FIG 3:**
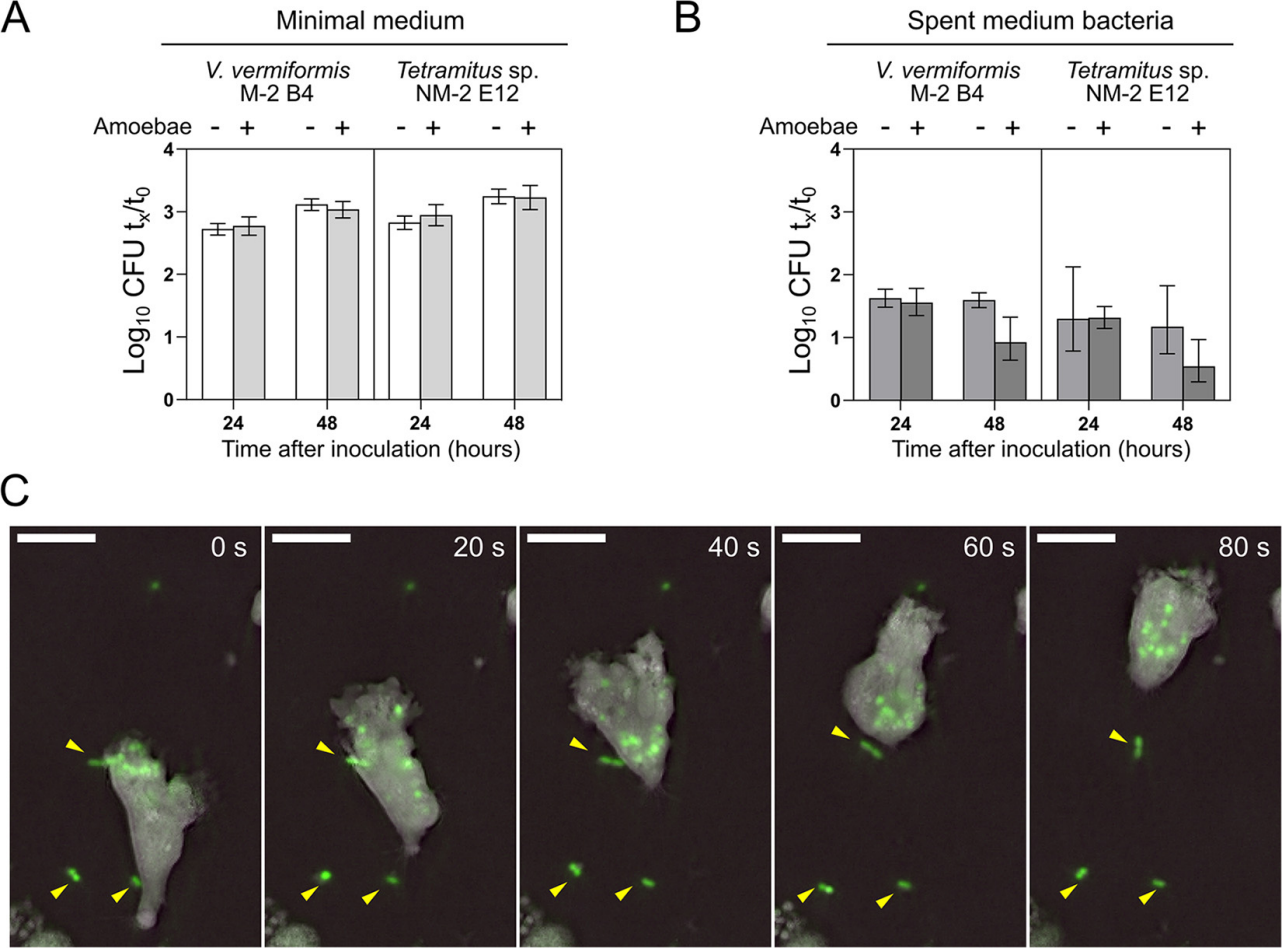
Bactericidal activity of isolated amoebae results from trophic interaction. (A) A. baumannii AB5075F was inoculated in minimal medium conditioned by V. vermiformis M-2 B4 or *Tetramitus* sp. NM-2 E12 for 24 h. (B) Same experiment as in panel A but with AB5075F inoculated in spent medium of bacteria (A. baumannii) alone or in coculture with V. vermiformis M-2 B4 or *Tetramitus* sp. NM-2 E12. For both panels A and B, CFU counts were determined at 0 h, 24 h, and 48 h, and growth is expressed as the ratio of the log_10_ CFU counts at 24 h and 48 h (*t_x_*) relative to the inoculated CFU count (*t*_0_). (C) Holotomographic microscopy of V. vermiformis M-2 B4 interacting with the GFP-expressing A. baumannii AB5075F (yellow arrowheads). Images were recorded using simultaneous holotomographic and epifluorescence imaging. Holotomographic RI values were recorded with a maximum temporal resolution of 0.5 three-dimensional RI volume per second. Snapshots were taken at 20-s intervals. Scale bars represent 10 μm.

Amoebae were isolated based on their ability to feed on A. baumannii or K. pneumoniae and thus are expected to internalize bacteria in a digestive vacuole by phagocytosis, which constitutes the basis of their bactericidal activity. However, the bactericidal activities of axenic isolates of V. vermiformis, Acanthamoeba polyphaga, A. castellanii, Acanthamoeba lenticulata, and D. discoideum toward the rice pathogen Xanthomonas oryzae were reported to essentially stem from the production of antibacterial compounds (49). X. oryzae was rarely observed within the digestive vacuoles of the amoebae (49). To determine whether the amoebae isolated in this study produced bactericidal or bacteriostatic compounds, we analyzed bacterial growth in culture supernatants of V. vermiformis M-2 B4 and *Tetramitus* NM-2 E12. To test this, bacteria were inoculated either in fresh medium or in the same medium previously incubated with V. vermiformis M-2 B4 or *Tetramitus* sp. strain NM-2 E12. Both bacterial strains showed similar growth in the fresh medium and in amoebal culture supernatants, indicating that the amoebae had not released bactericidal or bacteriostatic compounds (Fig. 3A). We then tested the possibility that amoebae produce these compounds only when presented with their bacterial prey. We thus tested the growth of A. baumannii in coculture supernatants of the bacterial strain and amoebae and in supernatants of the bacteria alone. In this situation, bacterial growth was limited because the carbon source had been previously exhausted. Again, however, no bactericidal or bacteriostatic effect of amoebae cocultivation could be detected after 24 h of exposure (Fig. 3B). At 48 h, a limited reduction was observed but was not statistically significant.

The absence of bactericidal activity in culture supernatants suggests that A. baumannii is killed while intracellular. However, our experimental determination of the fate of the A. baumannii population relied on viable counts on plates (CFU), and we could not exclude the possibility that the interaction with amoebae triggered a nonreplicative state of extracellular A. baumannii. To test this, we conducted an experiment in which the fate of A. baumannii in contact with V. vermiformis M-2 B4 and *Tetramitus* NM-2 E12 was followed both by CFU counting and by direct quantification of bacterial counts with flow cytometry. To do this, we used the GFP-expressing A. baumannii AB5075F to distinguish it from other particles or amoebal cells (see Fig. S2A). We found that the CFU counts were largely consistent with direct bacterial counts by flow cytometry, indicating that the bacterial cells physically disappear when in contact with the amoebae (see Fig. S2B). We then examined the fate of bacteria incubated with amoebae using microscopy. Transmission electron microscopy of a *Tetramitus* sp. isolate incubated with A. baumannii revealed the presence of particles resembling A. baumannii within vacuolar compartments (see Fig. S3A). Confocal fluorescence microscopy of *Tetramitus* incubated with GFP-expressing A. baumannii indeed confirmed numerous GFP-positive vacuoles (see Fig. S3B). We also used holotomographic imaging combined with epifluorescence imaging, in which GFP-positive spherical particles larger than bacteria were also observed to be associated with the highly motile V. vermiformis M-2 B4 (Fig. 3C). In contrast to extracellular bacteria (Fig. 3C, yellow arrowheads), these fluorescent compartments moved along with the amoebae, indicating an intracellular localization (Fig. 3C). Thus, the results of microscopic analyses are consistent with A. baumannii being phagocytosed in digestive vacuoles to support amoebal growth. We conclude that the observed bactericidal activity of the isolated amoebae is most likely due to their active feeding on bacteria.

### Most isolated amoebae display broad-spectrum bactericidal activity.

**FIG 4:**
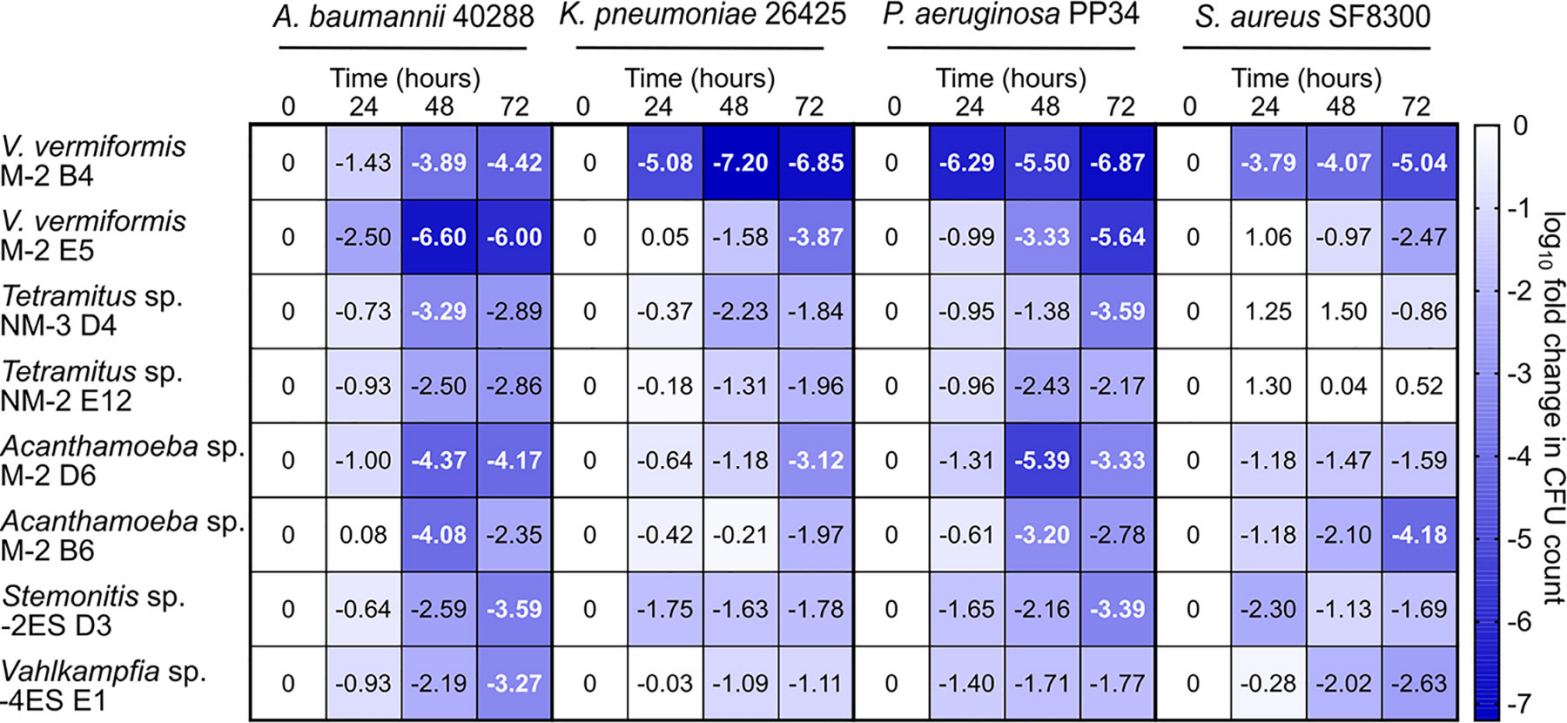
Isolated amoebae display broad-spectrum bactericidal activity against clinical isolates. Encysted amoebae (10^5^ cysts) were presented with clinical isolates (10^6^ CFU) on solid medium. CFU counts were determined at each time point and expressed as the log_10_ ratio of the bacterial population with and without amoebae. If no CFU were detected, then the sample was given the value of the detection limit (5 CFU).

The fact that the bactericidal activity resulted from trophic interaction suggests that the isolated amoebae may kill diverse species that they may ingest, including antibiotic-resistant clinical isolates. We thus challenged eight of the isolated amoebae with four antibiotic-resistant strains, i.e., A. baumannii 40288 (resistant to carbapenems), K. pneumoniae 26425 (resistant to cephalosporins), P. aeruginosa PP34 (resistant to carbapenems), and S. aureus (resistant to methicillin). Bacterial isolates (1 × 10^6^ CFU) were inoculated on solid medium with or without encysted amoebae (1 × 10^5^ cysts). The bacterial CFU counts for these two conditions were determined daily, and a fold change (FC) is reported as a log_10_ ratio (log_10_FC) (Fig. 4). Bactericidal activities were globally the highest toward A. baumannii 40288. For this clinical isolate, the presence of any of the tested amoebae reduced by 2 orders of magnitude the viable CFU count at 72 h (log_10_FC of >2). Most amoebae, with the exception of *Vahlkampfia* sp. strain 4ES E1, could also reduce the viable counts of P. aeruginosa PP34 by >100-fold (log_10_FC of >2) in 72 h. However, bactericidal activity was globally less important against K. pneumoniae 26425, as the log_10_FC was <2 for five of the eight amoebae at 72 h. For instance, *Vahlkampfia* sp. strain 4ES E1, which could reduce the viable counts of A. baumannii by 1,000-fold (log_10_FC of >3), could reduce the viable counts of K. pneumoniae by only 10-fold (log_10_FC of ∼1). The Gram-positive S. aureus proved more resistant than the three tested Gram-negative bacteria. However, *Acanthamoeba* sp. strain M-2 B6, V. vermiformis M-2 B4, V. vermiformis M-2 E5, and *Vahlkampfia* sp. strain 4ES E1 displayed log_10_FC values of >2. The two *Tetramitus* sp. isolates did not display any bactericidal activity against S. aureus (log_10_FC of ∼0), although they efficiently killed A. baumannii. Interestingly, V. vermiformis M-2 B4 showed the greatest bactericidal activities of all tested amoebae. It could reduce the viable counts of all tested species by >4 orders of magnitude at 72 h. Moreover, the bactericidal effect of this amoeba was also high at 24 h, reducing the viable counts of K. pneumoniae and P. aeruginosa PP34 by >5 orders of magnitude (Fig. 4). Overall, all tested amoebae were effective at killing bacteria other than those on which they were selected, as a source of food. With the exception of *Tetramitus*, they showed some killing activity against the Gram-positive S. aureus. Altogether, the results indicate that environmental amoebae have broad-spectrum bactericidal activity, the extent of which varies among amoebal species and isolates.

### V. vermiformis M-2 B4 is bactericidal to highly virulent clinical isolates.

**FIG 5:**
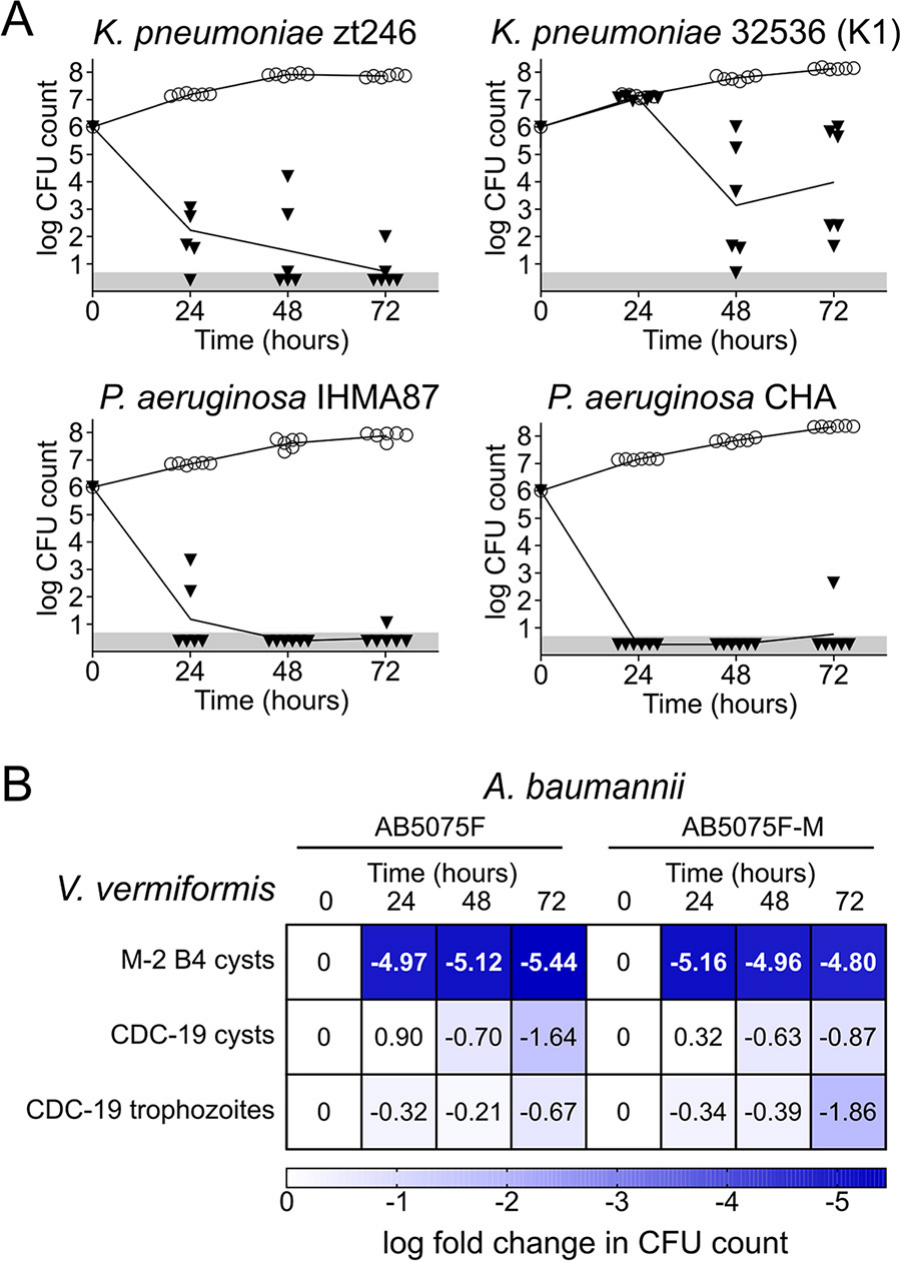
V. vermiformis M-2 B4 displays strong broad-spectrum bactericidal activity against clinical isolates. (A) V. vermiformis M-2 B4 kills clinical isolates of K. pneumoniae and P. aeruginosa. Isolates of K. pneumoniae and P. aeruginosa (∼10^6^ bacteria) were deposited alone (open circles) or exposed to V. vermiformis M-2 B4 (black inverted triangles). CFU counts at time zero are based on enumeration of the bacteria deposited in the well. CFU counts at 24, 48, and 72 h correspond to the number of bacteria recovered from the wells. If no CFU were detected, then the sample was given the value of the detection limit (5 CFU) (gray area), corresponding to the number of CFU possibly present in the nonplated fraction of the collected sample. (B) V. vermiformis M-2 B4 but not V. vermiformis CDC-19 (ATCC 50237) is bactericidal to A. baumannii. Amoebal cysts or trophozoites (10^5^ cells) were presented with A. baumannii AB5075F or AB5075F-M (10^6^ CFU) on solid medium. CFU counts were determined at each time point and expressed as the log_10_ ratio of the bacterial populations with and without amoebae. If no CFU were detected, then the sample was given the value of the detection limit (5 CFU).

We next tested V. vermiformis M-2 B4, the most bactericidal isolate, against additional clinical isolates of K. pneumoniae and P. aeruginosa (Fig. 5A). Confirming its potent bactericidal activity, M-2 B4 reduced the viability of two K. pneumoniae clinical isolates by 5 log units at 24 h. The clinical isolate 32536, a hypermucoviscous K1 capsular serotype that is considered hypervirulent (50) and resistant to phagocytosis by neutrophils (51), proved more resistant, with no reduction in viable counts at 24 h. At 48 h and 72 h, however, the viable counts in the presence of V. vermiformis M-2 B4 were lower by at least 2 orders of magnitude (Fig. 5A). V. vermiformis M-2 B4 was then tested against another two P. aeruginosa clinical isolates, including IHMA87, an exolysin-secreting clinical isolate with cytotoxicity to different eukaryotic cell lines (52, 53). V. vermiformis M-2 B4 brought the viable CFU counts of the two isolates under the detection limit at 48 h. The high bactericidal activities of V. vermiformis M-2 B4 prompted us to compare it to the widespread, axenically growing V. vermiformis CDC-19 (ATCC 50237) (54). While V. vermiformis M-2 B4 could consistently lower the viable A. baumannii AB5075 population by >5 orders of magnitude, V. vermiformis CDC-19 did not impact the viable counts, whether the predation assay was initiated with cysts (like M-2 B4) or already active trophozoites (Fig. 5B). This suggests that environmental amoebae have stronger bacterial activity than domesticated and axenically growing isolates.

## DISCUSSION

Here, we report the isolation of free-living amoebae from compost, a medium in which many microorganisms coexist and promote the breakdown of organic matter (55). With the objective of testing whether environmental free-living amoebae could predate on human pathogens, we isolated amoebae based on their ability to feed on A. baumannii and K. pneumoniae. The isolation of amoebae able to grow on K. pneumoniae led to the identification of the same three amoebal genera observed during the isolation campaign with A. baumannii. However, the proportions of each amoebal genus isolated on K. pneumoniae and A. baumannii differed. Prey size, the presence of surface molecular patterns, or hydrophobicity may influence the phagocytic capacity and the isolation of specific amoebae (56, 57). However, the difference in amoebal genus proportions isolated in the two screening campaigns and the specific isolation of *Vahlkampfia* sp. and *Stemonitis* sp. on K. pneumoniae likely do not reflect a differential ability of amoebae to better phagocyte one of the two bacterial species. Indeed, we subsequently found that amoebae isolated on A. baumannii could feed on K. pneumoniae and vice versa (Fig. 4). Rather, it is more likely that seasonal variations between the two screening campaigns could be the cause of the differences. The campaigns for isolations on A. baumannii and K. pneumoniae were conducted using samples from the same open-air compost site but 1 month apart, in January and February, respectively. While the weather in January 2019 in the Auvergne-Rhône Alpes region was cold and rainy, the end of February 2019 was warmer and dry, and variations in climatic conditions been observed to influence the abundance of different amoebal genera (5, 58, 59).

One of the features of pathogens proposed to limit predation by phagocytes is the production of an extracellular capsule. This was primarily supported in the fungus Cryptococcus neoformans, whose capsule protected it against A. castellanii (60). A protective role of the capsule against amoebal predation was also demonstrated in the case of K. pneumoniae against the social amoeba D. discoideum (18). Capsule production was found to shield S. aureus, K. pneumoniae, and Streptococcus pneumoniae from phagocytosis by mammalian phagocytes (61–64). The two K. pneumoniae clinical isolates selected as food sources displayed a mucoid phenotype on plates, as is expected for this species, which is known for expressing a thick capsule (65). A. baumannii also naturally displays a polysaccharidic capsule, but its production is stimulated by subinhibitory concentrations of antibiotics and increases bacterial virulence during infection (66). We thus included in our study a constitutively mucoid strain with a mutation (S551L) in the autokinase domain of *wzc* that causes a regulation defect in capsule production (66). Isolation of amoebae on this constitutively capsulated mutant of A. baumannii did not prove more challenging than that on the parental strain. Overall, the constitutive production of a thick polysaccharide capsule by the A. baumannii strain did not seem to impact the predation capacity and the kinetics of killing by the different amoebae (Fig. 2). This was rather unexpected, given the aforementioned reports that capsule production provided resistance to phagocytes. We cannot exclude the possibility that the difference in capsule production by the parental strain and the *wzc* mutant observed on the agar plates is not retained under amoebal predation. It is also possible that capsule production offers some relative protection, but amoebae can overcome it when the capsulated bacteria are their only food source. Consistent with this, V. vermiformis M-2 B4 could even predate on the hypermucoviscous capsular serotype K1 of K. pneumoniae, albeit less efficiently than on other K. pneumoniae isolates (Fig. 5). Thus, although capsule production can alter predation kinetics, it does not constitute a bulletproof vest against predatory amoebae.

Differences in the kinetics of bactericidal activity between isolates of different genera were noticeable (Fig. 2 and 4). For instance, one isolate of V. vermiformis was able to kill the bacterial population as early as 24 h, while no isolates of *Acanthamoeba* could show bactericidal activity before the 48-h time point. It should be noted that all amoebae were encysted at the time they were exposed to bacteria and the excystment time could vary between amoebae and was shorter for V. vermiformis than for *Acanthamoeba* (67, 68). While excystment into the first trophozoites was observed after approximately 9 h for V. vermiformis ATCC 50237 (68), we observed that a majority of cysts of V. vermiformis excysted as trophozoites in as little as 3 h and no cysts were observed at 6 h (see Fig. S4 in the supplemental material). Thus, amoebae that excysted faster could predate on bacteria sooner and reduce bacterial viability more rapidly. Some amoebae, such as the *Tetramitus* and *Vahlkampfia* isolates, displayed an initial ability to reduce the bacterial population by 10- to 100-fold but no further decline occurred over time, suggesting that predation stopped, establishing a form of equilibrium. Predation activity was found to be dependent on prey density for strains of *Tetramitus*, *Hartmanella*, *Naegleria*, and *Vahlkampfia* toward Rhizobium meliloti bacteria, possibly because feeding is limited by the ability to capture rare prey (69). The cessation of predation activity by these amoebae could also be linked to the production of molecules by P. aeruginosa and A. baumannii that force the amoebae to encyst (70) or lead to their death (71), respectively. P. aeruginosa may also kill amoebae using the type III secretion system (72). However, the two members of V. vermiformis displayed strong and sustained bactericidal activity, bringing A. baumannii populations down to the detection limit (Fig. 2). Indeed, although the axenic V. vermiformis laboratory strain CDC-19 could not predate on A. baumannii, the natural isolate M-2 B4 of the same species could efficiently kill this pathogen. Similarly, we observed that wild A. castellanii isolates could kill and feed on A. baumannii, which was previously reported to be resistant to laboratory strains of A. castellanii (71, 73). It may be that laboratory strains have diminished bactericidal activity. Laboratory-domesticated amoebae have been selected to grow axenically by feeding on liquid medium through micropinocytosis rather than by predating on bacteria through phagocytosis (74). Although this is reversible, axenic D. discoideum grows less efficiently on bacteria (75), indicating that axenic amoebae may be partly defective in predating on pathogens. However, high bactericidal activity could also be unique to specific isolates, such as V. vermiformis M-2 B4, which could eliminate K. pneumoniae and P. aeruginosa but also consume the Gram-positive pathogen S. aureus. V. vermiformis M-2 B4 could clear populations of P. aeruginosa, including the PP34 isolate, which contains the type III secretion system and produces the ExoU toxin (52) involved in killing A. castellanii (72, 76), and also the highly virulent CHA isolate, which can induce ExoU-independent oncosis in phagocytic cells (77) (Fig. 5). It is possible that the exceptionally high bactericidal activity of M-2 B4 is due to the fact that it is immune to mechanisms used by bacteria to mitigate predation by amoebae.

Importantly, M-2 B4 was found by characterizing only a subset of the 104 amoebae isolated in this study, and other isolates may display similar characteristics. While some amoebae seem to have intrinsically high predatory activities, it should be noted that we monitored the outcome of the interaction of amoebae and bacteria outside their natural environment, by offering amoebae a single bacterial source of nutrient. However, soil bacteria are expected to face complex bacterial communities and may discriminate and feed on specific bacteria (57). For instance, in an experimental soil system, A. castellanii was found to preferentially predate on *Betaproteobacteria* and *Firmicutes*, rather than on *Actinobacteria*, *Nitrospira*, *Verrucomicrobia*, or *Planctomycetes*, rapidly inducing shifts in the bacterial community composition (6). In the same line of thinking, although we found that capsulated and noncapsulated bacteria were both digested by wild amoebae, it remains possible that amoebae may preferentially consume the noncapsulated bacteria in more natural settings.

In conclusion, we report here that free-living amoebae capable of predating on human pathogens can be easily recovered from natural environments. Pathogens such as A. baumannii and K. pneumoniae, which were previously reported to be resistant to killing by domesticated *Acanthamoeba* strains (71, 73, 78), were easily consumed by natural isolates of the same species. Our work supports the idea that axenically growing laboratory amoebae poorly reflect the relationship of human pathogens and amoebae in natural environments, overestimating the chance of pathogen survival in the war against predatory amoebae. Rather, we propose that the natural environment is a rich source of diverse amoebae with broad-spectrum predatory activities against human pathogens, including against antibiotic-resistant isolates.

## MATERIALS AND METHODS

### Bacterial growth conditions and strains.

All bacterial isolates were cultivated in liquid or solid lysogeny broth (LB) or tryptic soy broth (TSB). Acinetobacter baumannii AB5075 is a human clinical isolate that is highly virulent in a mouse model (79) and is resistant to carbapenems (80). A. baumannii 40288 is an animal clinical isolate from the ST25 lineage and is resistant to carbapenems (81). AB5075F is a derivative of AB5075 that was naturally transformed with a synthetic PCR product to insert the genes encoding the superfolder GFP (*sfgfp*) and resistance to apramycin (82) at the *att*Tn*7* site downstream of the *glmS* gene (83). The 40288 strain used in this study was also naturally transformed to become resistant to apramycin. AB5075F-M is a naturally occurring mutant of AB5075F harboring a S551L mutation in the *wzc* gene. K. pneumoniae zt246 is sensitive to most classes of antibiotics (except first-generation β-lactams), while K. pneumoniae 26425 is resistant to cephalosporins (cefotaxime, ceftazidime, and cefuroxime), fluoroquinolones (ofloxacin and enrofloxacin), the aminoglycoside tobramycin, and sulfonamides. K. pneumoniae 32536 is a K1 capsular serotype strain that is sensitive to most antibiotics except the β-lactam amoxicillin. The hypermucoviscous phenotype of the K1 capsular serotype was confirmed by the formation of a string of >5 mm when an inoculation loop was stretched upward from colonies on an agar plate (84). P. aeruginosa PP34 is a clinical isolate that produces the cytotoxic exoenzyme ExoU and is resistant to the fluoroquinolones ciprofloxacin and moxifloxacin, to the cephalosporin cefepime, and to the carbapenem imipenem (85). P. aeruginosa CHA is a highly virulent clinical isolate with a mucoid phenotype (86). Staphylococcus aureus SF8300 is a USA300 clone and is resistant to methicillin, erythromycin, and cefotaxime (87).

### Isolation of environmental free-living amoebae feeding on A. baumannii or K. pneumoniae.

The samples used to isolate amoebae capable of feeding on A. baumannii or K. pneumoniae were collected from an open-air compost site in l’Arbresle, France. The first sample, which was used to isolate amoebae that could grow on A. baumannii, was collected in January 2019. Five grams of sample was mixed with 10 ml of Page’s amoeba saline (PAS) (2 mM NaCl, 0.016 mM MgSO_4_, 0,0.027 mM CaCl_2_, 0.79 mM Na_2_HPO_4_, 0.99 mM KH_2_PO_4_) for 5 min using a vortex mixer. Serial dilutions of the suspension were spotted on NNA (4.6 mM Na_2_HPO_4_, 2.9 mM KH_2_PO_4_, 15 g/liter bacteriological agar) coated with A. baumannii AB5075F lawns and were incubated for 7 days at 30°C. Equal numbers of petri dishes were coated with the A. baumannii AB5075F strain and with the constitutively capsulated A. baumannii AB5075F-M strain. Daily microscopic observations allowed isolation of emerging amoebae. Each isolated amoeba was then subcultured two more times on fresh NNA with the same bacterial lawn. Isolated amoebae were stored at −80°C in a mixture of peptone-yeast-glucose (ATCC medium 712) [0.05 M CaCl_2_·2H_2_O, 0.4 M MgSO_4_·7H_2_O, 0.25 M Na_2_HPO_4_·7H_2_O, 0.25 M KH_2_PO_4_, 0.1% sodium citrate dehydrate, 5 mM Fe(NH_4_)_2_(SO_4_)_2_·6H_2_O, 0.1 M glucose [pH 6.5]] and 10% dimethyl sulfoxide (DMSO). Isolation of amoebae capable of phagocytosing K. pneumoniae was performed with another sample collected from the same composting site at the end of February 2019. The new sample was processed as described above, and dilutions were spotted on NNA coated with K. pneumoniae zt246 or K. pneumoniae 26425. Amoebae able to grow on those bacteria were isolated and stored as described above.

### Identification of isolated amoebae.

To identify amoebae, 1-week cultures of each isolated amoeba were collected in 2 ml of 1× phosphate-buffered saline (PBS) (0.13 M NaCl, 8 mM Na_2_HPO_4_·2H_2_O, 0.18 mM KH_2_PO_4_, 2.7 mM KCl) and heated at 80°C for 10 min. Amplification of an ∼650-bp fragment of the 18S SSU rRNA region was carried out by PCR using specific primers F-566 (5′-CAGCAGCCGCGGTAATTCC-3′) and R-1200 (5′-CCCGTGTTGAGTCAAATTAAGC-3′) (88). PCR products were then purified using AMPure XP magnetic beads (Beckman Coulter, USA) and sequenced (Eurofins Genomics, Germany). Sequences were aligned in SeaView v5.0.4 (Pôle Rhône-Alpes de Bioinformatique Site Doua, Lyon, France) using the MUSCLE algorithm before being manually inspected. Reference sequences and representative sequences of different amoebal genera and *Acanthamoeba* genotypes were retrieved from the NCBI database. The accession numbers for all reference sequences are available in Table S1 in the supplemental material. Maximum likelihood tree construction was carried out with 437 selected sites of SSU ribosomal DNA (rDNA) using the PhyML v3.0 algorithm (89) of the ATGC Montpellier Bioinformatic Platform with a GTR model, optimized equilibrium frequencies, NNI tree improvement, and 1,000 bootstrap replicates.

### Quantification of amoebal predation activities against A. baumannii AB5075.

A. baumannii AB5075F and AB5075F-M were cultured in LB for 3 h and then washed twice in PBS. The suspensions were next adjusted to 10^8^ CFU/ml on the basis of absorbance, and the CFU counts were verified by plating. Ten microliters of each bacterial suspension (∼10^6^ bacteria) was spotted at the center of a well of a 24-well plate containing 2 ml of NNA-Gelrite (NNA with 10 g/liter Gelrite [Carl Roth, Germany]) and then dried. Amoebal isolates were cultured on NNA on a lawn of E. coli K-12 bacteria for 7 days at 30°C. Each amoeba was then collected, washed twice in PBS, and incubated overnight in a PBS solution containing penicillin-streptomycin (1,000 units/ml penicillin and 1 mg/ml streptomycin; Thermo Fisher Scientific, USA). Amoebal suspensions were then washed twice with PBS and starved for 1 week in Neff’s encystment medium (NEM) (containing, in 1 liter of distilled water, 0.1 M KCl, 0.39 mM MgSO_4_, 0.3 mM CaCl_2_, 0.9 mM NaHCO_3_, and 0.2 mM 2-amino-2-methyl-1,3-propanediol [pH 8.8 to 9]). On the day of the experiment, suspensions of amoebae were washed twice in PBS and then diluted to obtain a concentration of 10^7^ cysts/ml. Ten microliters of each suspension was spotted on top of the previously spotted bacteria in the 24-well plate. Plates were then incubated for 72 h at 30°C, and the contents of the wells were recovered by adding 150 μl of PBS and two or three glass beads, followed by gentle shaking. The liquid (∼100 μl) was recovered, and serial dilutions were plated on LB medium containing apramycin at 30 μg/ml to determine CFU counts. Each experiment consists of three bacterium-cyst mixtures (from three independent bacterial cultures), for which two replicates were used to determine CFU counts. The experiments presented were conducted at least twice.

### Quantification of amoebal predation against multiple antibiotic-resistant clinical isolates.

Clinical isolates of human pathogens were grown in LB (A. baumannii, K. pneumoniae, and P. aeruginosa) or TSB (S. aureus). Cultures were washed in PBS and diluted to 10^8^ CFU/ml as described above. Ten microliters of each bacterial suspension was placed in 24-well plates containing 2 ml of NNA-Gelrite and then dried. Selected isolates of amoebae were cultured, starved, and adjusted to 10^7^ amoebae/ml as described above. Ten microliters of each suspension was spotted on top of the bacteria. Plates were then incubated for 72 h at 30°C, and the contents of the wells were collected as described above to determine CFU counts. The viability of A. baumannii 40288 was evaluated by plating on LB medium containing apramycin at 30 μg/ml. Isolates of K. pneumoniae and P. aeruginosa were plated and counted on LB medium containing ampicillin at 50 μg/ml, while the S. aureus isolate was plated and counted on tryptic soy agar (TSA) containing ampicillin at 50 μg/ml.

### Effect of amoebal supernatant on bacterial viability.

A. baumannii AB5075F was incubated (10^6^ CFU/ml) at 30°C for 48 h in minimal acetate medium (MAM) [0.07 M KH_2_PO_4_, 0.03 M Na_2_HPO_4_, 0.02 M (NH_4_)_2_SO_4_, 0.8 mM MgSO_4_, 0.007 mM CaCl_2_, 0,004 mM FeSO_4_ and 1 g/liter sodium acetate] alone as a control or coinoculated with encysted *Vermamoeba* M-2 B4 or *Tetramitus* NM-2 E12 (10^5^ amoebae/ml). The amoebae tested were also inoculated (10^5^ amoebae/ml) alone under the same conditions. The different cultures were then centrifuged gently (600 × *g* for 10 min) to prevent cell lysis. Bacteria were then inoculated (10^6^ CFU/ml) at 30°C in the resultant filtered supernatants (0.2-μm Acrodisc; Pall Corp., USA) from different culture conditions or in fresh MAM. After 24 h and 48 h of incubation, the suspensions were plated at 37°C on LB medium containing apramycin at 30 μg/ml to determine CFU counts.

### Flow cytometric analysis.

Interactions between amoebae and AB5075F were set up as described above. At 0 h, 24 h, 48 h, and 72 h, the amoeba-bacterium mixture was recovered in PBS and fixed with formaldehyde (final concentration, 3.7%); membranes were stained with FM4-64 (final concentration, 10 μg/ml) for 30 min at room temperature and then washed twice with PBS. An Attune acoustic focusing cytometer (Life Technologies) was used for all flow cytometric acquisitions. Samples were run at a collection rate of 25 μl/min, and fluorescence emission was detected using a 530-nm/30-nm bandpass filter for GFP fluorescence and a 640-nm long-pass filter for FM4-64 fluorescence. The bacterial population was determined to be positive for GFP fluorescence and FM4-64 fluorescence. Particle counts were determined using the Attune software.

### Microscopic observations of amoeba-bacterium interactions. (i) Confocal microscopy.

*Tetramitus* sp. and AB5075F amoebae were processed as for the quantification of amoebal predation. Contents of the wells were recovered in 100 μl of PBS and deposited between a slide and a slipcover to be observed under confocal microscopy using a DMI4000 inverted microscope (Leica Microsystems, Germany) equipped with a W1 spinning-disk confocal head (Yokogawa, Japan).

### (ii) Transmission electron microscopy.

*Tetramitus* sp. amoebae were observed after cocultivation with A. baumannii AB5075F and suspended in 100 μl of PBS, centrifuged, and then fixed for 15 min in 0.2 M sodium cacodylate-4% glutaraldehyde solution. The fixed cell suspensions were then washed in 0.2 M cacodylate, embedded in 2% agar, and placed in 1% osmium tetroxide solution for 1 h. The samples were placed in contact with a 1% uranyl acetate solution for 1 h and then progressively dehydrated by placement in ethanol baths of increasing concentration for 10 min. The samples were then embedded in Epon resin. Ultrathin sections were prepared with an Ultracut UCS ultramicrotome (Leica Microsystems, USA), stained with a uranyl acetate-citrate solution, and then observed with a CM120kV transmission electron microscope (Philips, The Netherlands).

### (iii) Holotomographic imaging.

Holotomographic imaging was used in combination with epifluorescence imaging and was performed as described previously (90). V. vermiformis M-2 B4 was observed when fed with AB5075F, on a three-dimensional Cell Explorer-fluo (Nanolive, Ecublens, Switzerland) using a 60× air objective (numerical aperture, 0.8) at a wavelength of 520 nm (class 1 low-power laser; sample exposure, 0.2 mW/mm2) and a USB 3.0 CMOS Sony IMX174 sensor, with quantum efficiency (typical) of 70% (at 545 nm), dark noise (typical) of 6.6 e^−^, dynamic range (typical) of 73.7 dB, field of view of 90 by 90 by 30 μm, axial resolution of 400 nm, and maximum temporal resolution of 0.5 three-dimensional refractive index (RI) volume per second. The theoretical sensitivity was 2.71 × 10^−4^.

### Data availability.

Sequences were deposited in GenBank and are available under accession numbers MZ338393 to MZ338496, as listed in Table S2 in the supplemental material.
