# Supplementary material for: Environmental Free-Living Amoebae Can Predate on Diverse Antibiotic-Resistant Human Pathogens
Source: Appl Environ Microbiol. 2021 Aug 26;87(18):e00747-21. doi: 10.1128/AEM.00747-21 (PMC8388808; doi:10.1128/AEM.00747-21)
Supplement: Supplemental file 1 — Figures S1 to S4, Tables S1 and S2. Download AEM.00747-21-s0001.pdf, PDF file, 5.8 MB [file aem.00747-21-s0001.pdf]

Environmental free-living amoebae can predate on diverse antibiotic-resistant human pathogens

Running title: Amoebae predating on human pathogens

Félix Bornier[1], Eline Zas[1], Damien Potheret[1], Maria-Halima Laaberki[1], Bénédicte Coupat-Goutaland[1#*], Xavier Charpentier[1#*]

[1] CIRI, Centre International de Recherche en Infectiologie, Team Horizontal gene transfer in bacterial pathogens, Inserm, U1111, Université Claude Bernard Lyon 1, CNRS, UMR5308, École Normale Supérieure de Lyon, Univ Lyon, 69100, Villeurbanne, France

[#] These authors contributed equally

* Correspondance: benedicte.coupat-goutaland@univ-lyon1.fr, xavier.charpentier@univ-lyon1.fr

Supporting information

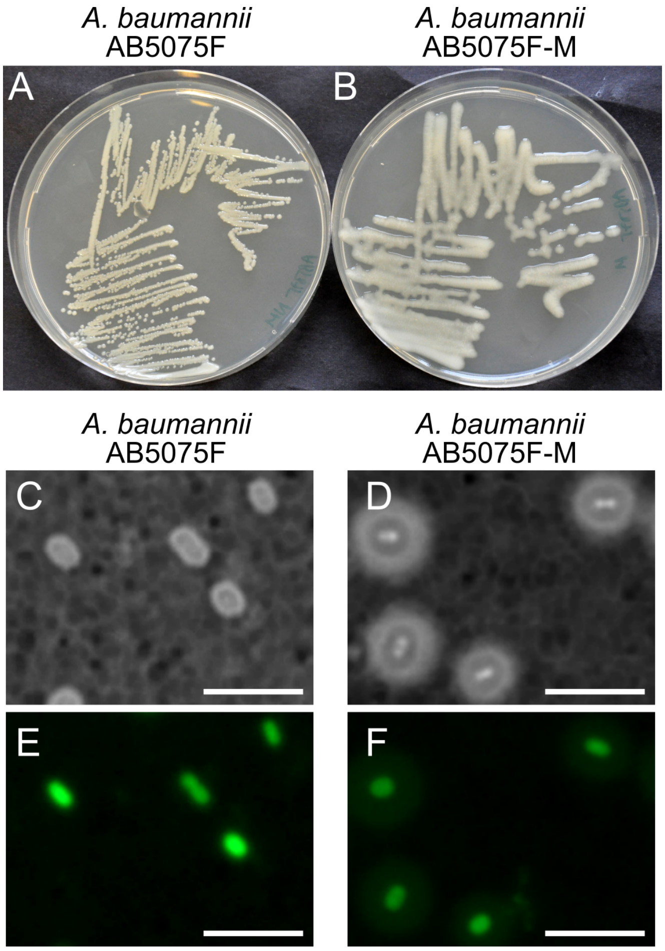

Figure S1. Identification of AB5075F-M, a mucoid mutant of *A. baumannii* AB5075F.

A and B, the mutant forms highly mucoid colonies on LB plates, compared to the parental strain. C and D, indian ink staining reveals a thick capsule (clear exclusion zone) around the cells. E and F, epifluorescence microscopy delineating the cytoplasm. Scale bar represent 10 µm.

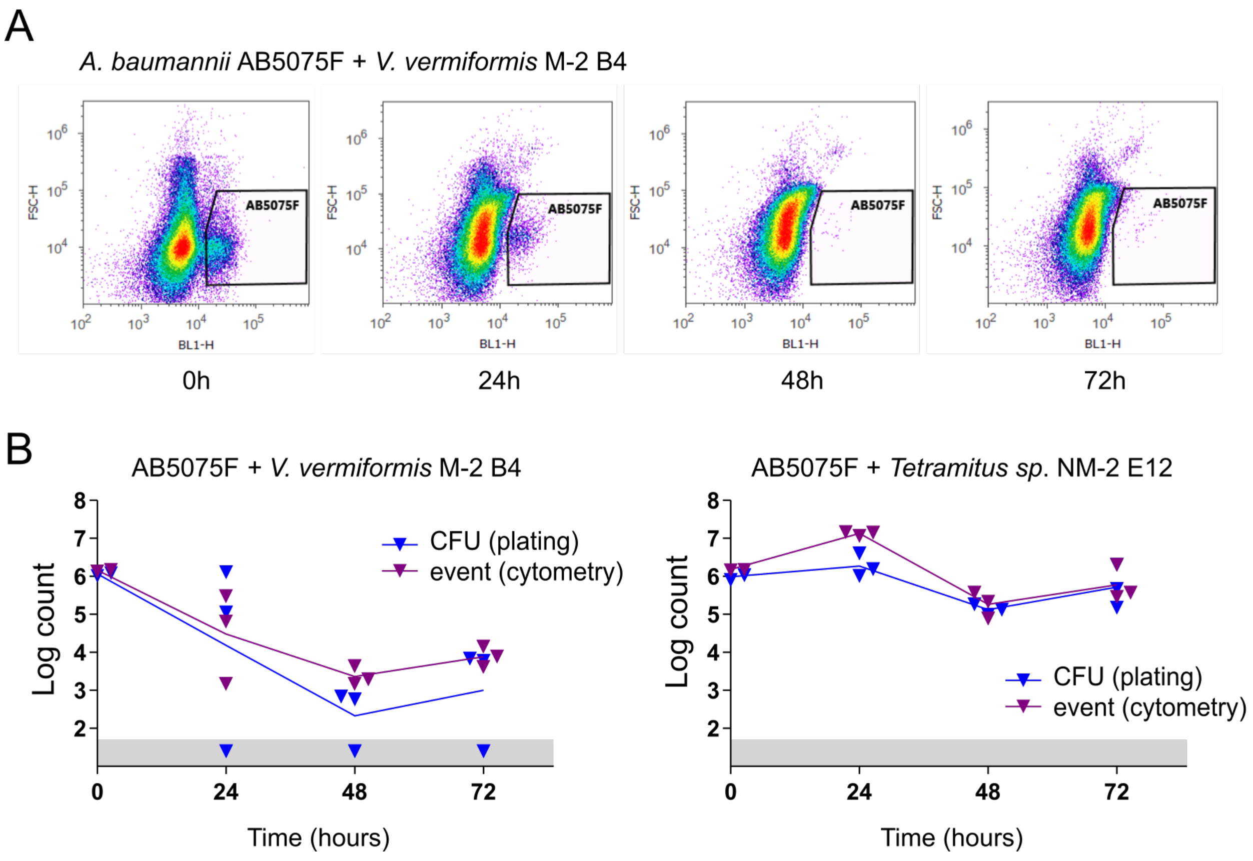

Figure S2. Flow cytometry analyses of amoebae/bacteria interactions

A. Flow cytometry analysis of *A. baumannii* AB5075F incubated with *V. vermiformis* M2 B4. AB5075F bacteria were detected on the basis of the expression of GFP (BL1-H axis), staining with the membrane dye FM4-64 (not shown) and the diffraction signal on the forward scatter (FSC-H axis).

B. Quantification of AB5075F upon incubation with *V. vermiformis* M2 B4 and *Tetramitus sp.* NM-2 E12 using viable counts (CFUs) and direct detection by flow cytometry. Both values were expressed as CFUs or event per mL of the resuspended mix of amoeba and bacteria.

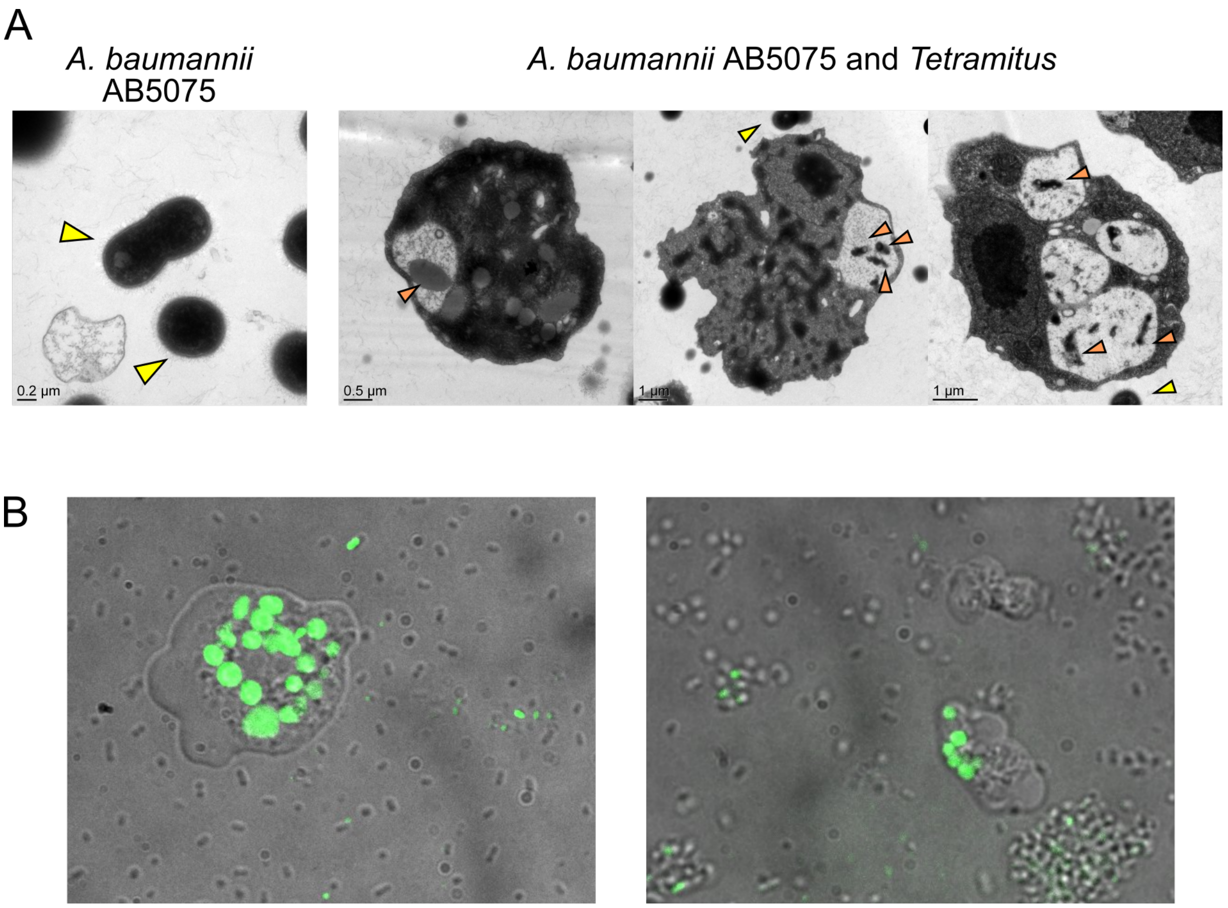

Figure S3. Visualization of *A. baumannii* within vacuoles of *Tetramitus*.

A. Transmission electron micrograph of *A. baumannii* alone (left) and a *Tetramitus* isolate incubated with *A. baumannii* as sole source of nutrient. Intact bacteria, or partly degraded in digestive vacuoles are indicated as yellow and orange arrowheads, respectively. Magnification varies in the micrographs and the horizontal black bar indicates the scale.

B. Confocal fluorescence microscopy of a *Tetramitus* isolate incubated with *A. baumannii* expressing sfGFP. Fluorescence images were overlayed on bright field images. Numerous vacuoles of *Tetramitus* are filled with fluorescent material. The focal plane is set on the vacuoles and the outside bacteria in the same focal plane are also be fluorescent.

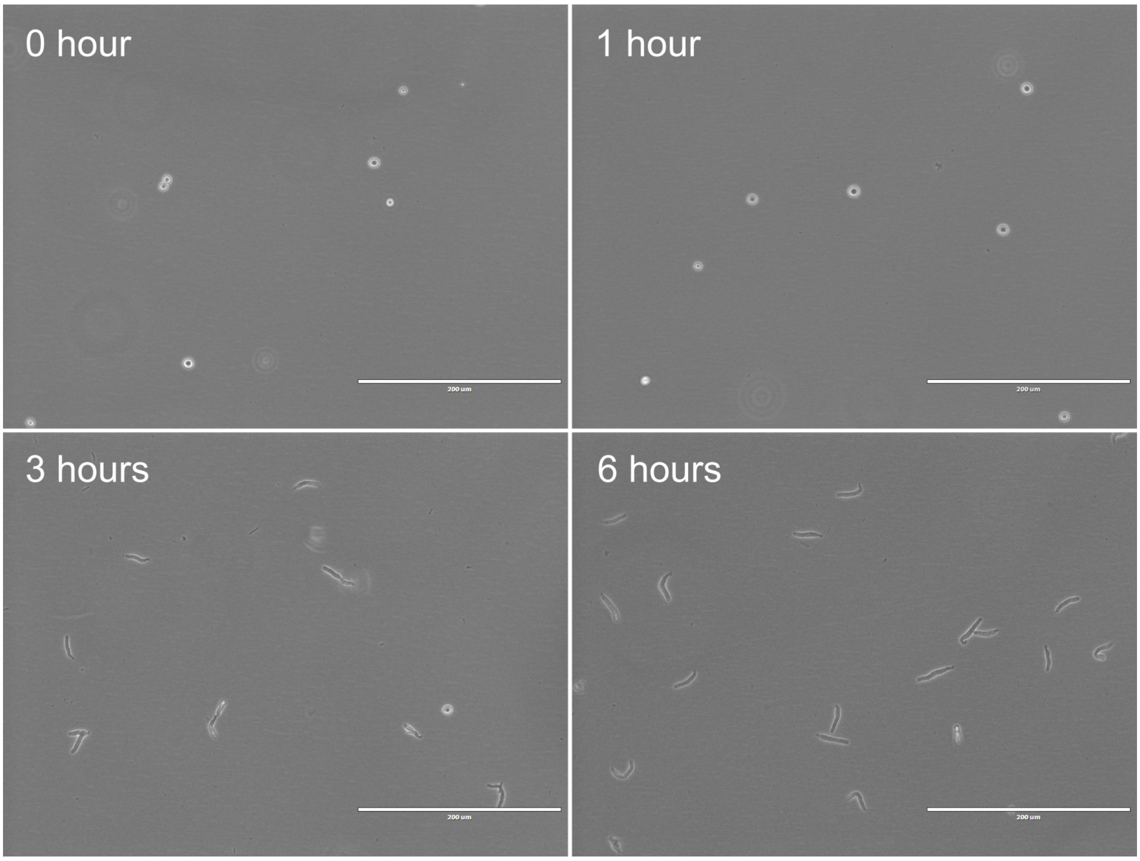

Figure S4. Excystment rate of *V. vermiformis* M-2 B4. Cysts were placed in PYNFH modified medium (ATCC 1034) and observed using bright field microscopy at 0, 1, 3 and 6 hours. Cysts present a rounded shape while trophozoites show an elongated form.

Table S1. Amoebae SSU-rDNA sequences used as reference in the phylogenetic tree and retrieved from the NCBI database. AK: *Acanthamoeba* keratitis, A: Animal, CC: cell culture, CLC: contact lens case, E: environmental strain, GAE: granulomatous amoebic encephalitis, HCC: human culture cell, HF: human facilities, HFe: human feces, HUr: human urine NM: nasal mucosa, S: stool,. Aul. Australia, Aus. Austria, Bar. Barbados, Bra. Brazil, Chi. China, Egy. Egypt, Fra. France, Ger. Germany, Isr. Israel, Ita. Italy, Jap. Japan, Kor. Korea, Mya. Myanmar, Pak. Pakistan, Rus. Russia, Slo. Slovenia, Slv. Slovakia, Swi. Switzerland, Thai. Thailand, Tun. Tunisia, UK. United Kingdom.

Genus	Strain	GenBank accession number	Genotype	Origin
Acanthamoeba sp.	*Acanthamoeba castellanii* Castellanii ATCC 50374	U07413	T4	CC, UK
	Acanthamoeba castellanii CDC:0981:V006	U07400	T1	GAE, USA
	Acanthamoeba castellanii Neff ATCC 50373	U07416	T4	E, USA
	Acanthamoeba sp. E_5C	AB425955	T2	E, Ita
	Acanthamoeba griffini S-7 ATCC 30731	U07412	T3	E, USA
	Acanthamoeba polyphaga Panola Mountain	AF019052	T3	E, USA
	Acanthamoeba royreba Oak Ridge ATCC 30884	U07417	T4	HCC, USA
	Acanthamoeba hatchetti 2HH	AF260722	T4	AK, Aus
	Acanthamoeba lenticulata PD2S	U94741	T5	E, Fra
	Acanthamoeba palestinensis 2802	AF019063	T6	E, Fra
	Acanthamoeba sp. EFW5	DQ992178	T7	E, Egy
	Acanthamoeba tubiashi OC-15C	AF019065	T8	E, USA
	Acanthamoeba sp. Ac_E2a	GU808280	T9	E, Thai
	Acanthamoeba culbertsoni Lilly A-1	AF019067	T10	HCC, USA
	Acanthamoeba hatchetti BH-2	AF019068	T11	E, USA
	Acanthamoeba healyi	AF019070	T12	GAE, Bar
	Acanthamoeba sp. UWET39	AF132136	T13	E, Ger
	Acanthamoeba sp. PN13	AF333609	T14	S, Pak
	Acanthamoeba jacobsi AC080	AY262361	T15	E, Aul
	Acanthamoeba sp. U/H-C1	AY026245	T16	AK, Bra
	Acanthamoeba sp. Ac_E9b	GU808302	T17	E, Thai
	Acanthamoeba rhysodes	AY351644	T4	E, UK
	Acanthamoeba mauritaniensis	AY351647	T4	E, UK
Stemonitis sp. and others Mixogastria	*Didymium* sp. E2/8	AY321111	-	E, Ger
	Didymium sp. E3P	AY321112	-	E, Ger
	Physarum flagellatum (Alexeieff)	AF411289	-	E, Rus
	Stemonitis aff. *flavogenita*	AF093247	-	HFe, Pak
	Stemonitis aff. *flavogenita* B1/2	AY321109	-	HFe, Ger
	Stemonitis flavogenita	AF239229	-	E, -
	Stemonitis flavogenita ATCC	HE614592	-	E, USA

	24714			
	Stemonitis herbatica HK0208001	MK041081	-	-
Tetramitus sp.	*Tetramitus dokdoensis*	KY463322	-	E, Kor
	Tetramitus entericus CCAP 1588/5	AJ224889	-	-
	Tetramitus rostratus	M98051	-	HUr, USA
	Tetramitus sp. BD1-1	EF378691	-	A, USA
	Tetramitus sp. BD1-5	EF378693	-	A, USA
	Tetramitus waccamawensis	AF011455	-	E, USA
Vahlkampfia sp.	*Vahlkampfia avara* CCAP 1588/1A	AJ224886	-	E, USA
	Vahlkampfia avara 4171L	JQ271723	-	-
	Vahlkampfia inornata CCAP 1588/2	AJ224887	-	E, USA
Vermamoeba sp.	*Vermamoeba vermiformis*	KY476315	-	-
	Vermamoeba vermiformis CCAP 1534/16	KC161965	-	HF, UK
	Vermamoeba vermiformis CCAP 1534/16	KC188996	-	HF, UK
	Vermamoeba vermiformis M.ut.1	KX856373	-	HF, Mya
	Vermamoeba vermiformis Pugl93F	KP792389	-	E, It
	Vermamoeba vermiformis Pugl97TW	KP792392	-	E, It
	Vermamoeba vermiformis Pugl102TW	KP792394	-	E, It
	Vermamoeba vermiformis Pugl104F	KP792396	-	E, It
	Vermamoeba vermiformis Pugl105F	KP792397	-	E, It
	Vermamoeba vermiformis TW EDP 1	KT266863	-	E, Fra
	Vermamoeba vermiformis TW EDP 3	KT266865	-	E, Fra
External group	*Nuclearia simplex*	AF484687	-	-, Ger

Table S2. Amoebae isolated from compost soil in this study and NCBI accession number of the SSU-rDNA sequences used in the phylogenetic tree.

Amoebae genera	Amoebae strains	Screening	Sampling period	NCBI accession number
Acanthamoeba sp.	NM-3 E3	*A. baumannii* AB5075F	January 2019	MZ338414
	M-2 B6	*A. baumannii* AB5075F-M	January 2019	MZ338411
	M-2 D5	*A. baumannii* AB5075F-M	January 2019	MZ338412
	M-2 D6	*A. baumannii* AB5075F-M	January 2019	MZ338413
	M-3 B1	*A. baumannii* AB5075F-M	January 2019	MZ338416
	M-3 B2	*A. baumannii* AB5075F-M	January 2019	MZ338415
	M-3 B3	*A. baumannii* AB5075F-M	January 2019	MZ338417
	-2WT A1	*K. pneumoniae* zt246	February 2019	MZ338418
	-2WT A3	*K. pneumoniae* zt246	February 2019	MZ338419
	-2WT A8	*K. pneumoniae* zt246	February 2019	MZ338420
	-2WT C2	*K. pneumoniae* zt246	February 2019	MZ338421
	-2WT C5	*K. pneumoniae* zt246	February 2019	MZ338422
	-2WT E5	*K. pneumoniae* zt246	February 2019	MZ338423
	-2WT E8	*K. pneumoniae* zt246	February 2019	MZ338424
	-3WT E1	*K. pneumoniae* zt246	February 2019	MZ338425
	-4WT C1	*K. pneumoniae* zt246	February 2019	MZ338426
	-4WT C2	*K. pneumoniae* zt246	February 2019	MZ338427
	-4WT C6	*K. pneumoniae* zt246	February 2019	MZ338428
	-2ES D10	*K. pneumoniae* 26425	February 2019	MZ338429
	-2ES E4	*K. pneumoniae* 26425	February 2019	MZ338430
	-3ES C1	*K. pneumoniae* 26425	February 2019	MZ338431
	-3ES C2	*K. pneumoniae* 26425	February 2019	MZ338432
	-3ES C4	*K. pneumoniae* 26425	February 2019	MZ338433
	-3ES C5	*K. pneumoniae* 26425	February 2019	MZ338434
	-3ES C6	*K. pneumoniae* 26425	February 2019	MZ338435
	-3ES C7	*K. pneumoniae* 26425	February 2019	MZ338436
	-3ES C8	*K. pneumoniae* 26425	February 2019	MZ338437
	-3ES D3	*K. pneumoniae* 26425	February 2019	MZ338438
	-3ES D4	*K. pneumoniae* 26425	February 2019	MZ338439
Stemonitis sp.	-2ES D3	*K. pneumoniae* 26425	February 2019	MZ338495
	-2ES E9	*K. pneumoniae* 26425	February 2019	MZ338496
Tetramitus sp.	NM-2 A2	*A. baumannii* AB5075F	January 2019	MZ338453
	NM-2 A5	*A. baumannii* AB5075F	January 2019	MZ338443
	NM-2 A6	*A. baumannii* AB5075F	January 2019	MZ338479
	NM-2 A8	*A. baumannii* AB5075F	January 2019	MZ338450
	NM-2 A10	*A. baumannii* AB5075F	January 2019	MZ338444
	NM-2 A12	*A. baumannii* AB5075F	January 2019	MZ338457
	NM-2 A13	*A. baumannii* AB5075F	January 2019	MZ338472
	NM-2 B2	*A. baumannii* AB5075F	January 2019	MZ338451
	NM-2 C3	*A. baumannii* AB5075F	January 2019	MZ338458
	NM-2 C6	*A. baumannii* AB5075F	January 2019	MZ338454
	NM-2 C8	*A. baumannii* AB5075F	January 2019	MZ338441
	NM-2 C10	*A. baumannii* AB5075F	January 2019	MZ338484
	NM-2 D3	*A. baumannii* AB5075F	January 2019	MZ338474

	NM-2 D7	*A. baumannii* AB5075F	January 2019	MZ338448
	NM-2 D9	*A. baumannii* AB5075F	January 2019	MZ338447
	NM-2 D11	*A. baumannii* AB5075F	January 2019	MZ338469
	NM-2 E2	*A. baumannii* AB5075F	January 2019	MZ338459
	NM-2 E3	*A. baumannii* AB5075F	January 2019	MZ338477
	NM-2 E4	*A. baumannii* AB5075F	January 2019	MZ338462
	NM-2 E6	*A. baumannii* AB5075F	January 2019	MZ338446
	NM-2 E11	*A. baumannii* AB5075F	January 2019	MZ338475
	NM-2 E12	*A. baumannii* AB5075F	January 2019	MZ338467
	NM-3 B3	*A. baumannii* AB5075F	January 2019	MZ338468
	NM-3 D4	*A. baumannii* AB5075F	January 2019	MZ338461
	NM-3 E1	*A. baumannii* AB5075F	January 2019	MZ338482
	NM-3 E4	*A. baumannii* AB5075F	January 2019	MZ338460
	NM-4 D1	*A. baumannii* AB5075F	January 2019	MZ338483
	M-2 A1	*A. baumannii* AB5075F-M	January 2019	MZ338481
	M-2 A3	*A. baumannii* AB5075F-M	January 2019	MZ338465
	M-2 A4	*A. baumannii* AB5075F-M	January 2019	MZ338464
	M-2 A7	*A. baumannii* AB5075F-M	January 2019	MZ338442
	M-2 A11	*A. baumannii* AB5075F-M	January 2019	MZ338463
	M-2 B1	*A. baumannii* AB5075F-M	January 2019	MZ338487
	M-2 B5	*A. baumannii* AB5075F-M	January 2019	MZ338486
	M-2 C2	*A. baumannii* AB5075F-M	January 2019	MZ338480
	M-2 C5	*A. baumannii* AB5075F-M	January 2019	MZ338449
	M-2 D1	*A. baumannii* AB5075F-M	January 2019	MZ338473
	M-2 E3	*A. baumannii* AB5075F-M	January 2019	MZ338452
	M-2 E4	*A. baumannii* AB5075F-M	January 2019	MZ338478
	M-3 A1	*A. baumannii* AB5075F-M	January 2019	MZ338455
	M-3 C2	*A. baumannii* AB5075F-M	January 2019	MZ338456
	M-3 C3	*A. baumannii* AB5075F-M	January 2019	MZ338476
	M-3 D1	*A. baumannii* AB5075F-M	January 2019	MZ338485
	M-3 D2	*A. baumannii* AB5075F-M	January 2019	MZ338445
	M-3 E1	*A. baumannii* AB5075F-M	January 2019	MZ338471
	M-3 E2	*A. baumannii* AB5075F-M	January 2019	MZ338440
	M-3 E3	*A. baumannii* AB5075F-M	January 2019	MZ338466
	M-3 E5	*A. baumannii* AB5075F-M	January 2019	MZ338470
	-2WT B1	*K. pneumoniae* zt246	February 2019	MZ338488
Vahlkampfia sp.	-2WT D8	*K. pneumoniae* zt246	February 2019	MZ338489
	-2ES D1	*K. pneumoniae* 26425	February 2019	MZ338490
	-4ES E1	*K. pneumoniae* 26425	February 2019	MZ338491
	-4ES E2	*K. pneumoniae* 26425	February 2019	MZ338492
	-4ES E3	*K. pneumoniae* 26425	February 2019	MZ338493
	-4ES E4	*K. pneumoniae* 26425	February 2019	MZ338494
Vermamoeba vermiformis	M-2 B4	*A. baumannii* AB5075F-M	January 2019	MZ338393
	M-2 E5	*A. baumannii* AB5075F-M	January 2019	MZ338394
	-2WT A4	*K. pneumoniae* zt246	February 2019	MZ338395
	-2WT A5	*K. pneumoniae* zt246	February 2019	MZ338396
	-2WT A10	*K. pneumoniae* zt246	February 2019	MZ338397
	-2WT E1	*K. pneumoniae* zt246	February 2019	MZ338398
	-2WT E7	*K. pneumoniae* zt246	February 2019	MZ338399
	-3WT A2	*K. pneumoniae* zt246	February 2019	MZ338400

	-3WT A5	*K. pneumoniae* zt246	February 2019	MZ338401
	-2ES B2	*K. pneumoniae* 26425	February 2019	MZ338402
	-2ES D6	*K. pneumoniae* 26425	February 2019	MZ338403
	-2ES D7	*K. pneumoniae* 26425	February 2019	MZ338404
	-2ES D8	*K. pneumoniae* 26425	February 2019	MZ338405
	-2ES E7	*K. pneumoniae* 26425	February 2019	MZ338406
	-4ES A1	*K. pneumoniae* 26425	February 2019	MZ338407
	-4ES A2	*K. pneumoniae* 26425	February 2019	MZ338408
	-4ES A3	*K. pneumoniae* 26425	February 2019	MZ338409
	-4ES A4	*K. pneumoniae* 26425	February 2019	MZ338410
